# Novel hyaluronic acid–methotrexate conjugate suppresses joint inflammation in the rat knee: efficacy and safety evaluation in two rat arthritis models

**DOI:** 10.1186/s13075-016-0971-8

**Published:** 2016-04-01

**Authors:** Tatsuya Tamura, Yoshinobu Higuchi, Hidetomo Kitamura, Naoaki Murao, Ryoichi Saitoh, Tadashi Morikawa, Haruhiko Sato

**Affiliations:** Research Division, Chugai Pharmaceutical Co., Ltd., 1-135 Komakado, Gotemba, Shizuoka 412-8513 Japan; New Business Planning Department, Denka Co., Ltd., 2-1-1 Nihonbashi-Muromachi, Chuo-ku, Tokyo, 103-8338 Japan

**Keywords:** Methotrexate, Hyaluronic acid, Chemical conjugate, Rheumatoid arthritis, Osteoarthritis, Intra-articular injection, Antigen-induced arthritis, Collagen-induced arthritis

## Abstract

**Background:**

Methotrexate (MTX) is one of the most widely used medications to treat rheumatoid arthritis (RA), and recent studies have also suggested the potential benefit of the drug for the treatment of osteoarthritis (OA) of the knee. MTX is commonly administered in oral formulations, but is often associated with systemic adverse reactions. In an attempt to address this issue, we have shown previously that a conjugate of hyaluronic acid (HA) and MTX exhibits potential as a drug candidate for intra-articular treatment of inflammatory arthritis. In this study, we compare the efficacy and safety of an optimized HA-MTX conjugate, DK226, with that of MTX in inflammatory arthritis rat models.

**Methods:**

In vitro activity of DK226 was assessed in human fibroblast-like synoviocytes (HFLS) and a synovial sarcoma cell line, SW982. Release of MTX from DK226 was investigated after incubation with rabbit synovial tissue homogenate or synovial fluid. In vivo efficacy of DK226 was evaluated in antigen-induced arthritis (AIA) and collagen-induced arthritis (CIA) in the rat knee. Pharmacokinetics and hematological toxicity after treatment with oral MTX or an intra-articular injection of DK226 were compared in AIA.

**Results:**

Proliferation of HFLS and SW982 cells was inhibited by DK226, and the inhibitory activity was reversed by cotreatment with excess HA or anti-CD44 antibody. MTX was released from DK226 by incubation with rabbit synovial tissue homogenate or synovial fluid at pH 4.0, but not at pH 7.4. AIA was ameliorated by intra-articular DK226, but not by HA, as potently as oral MTX. Hematological toxicity was induced by oral MTX, but not by DK226. The maximum plasma concentration of MTX after oral MTX was 40 times higher than the concentration of MTX after an intra-articular injection of DK226. Knee swelling in AIA was inhibited by intra-articular injections of DK226, but not by free MTX or a mixture of HA and MTX. In CIA, an injection of DK226 into the right knee joint significantly reduced swelling and synovial inflammation of the treated knee joint, but had no effect on the untreated contralateral knee joint.

**Conclusions:**

DK226 exerted anti-arthritic effects in two different models of arthritis. The conjugate had a wider therapeutic window than oral MTX, and could be a future drug for treatment of arthritic disorders, including inflammatory OA.

## Background

Rheumatoid arthritis (RA) and osteoarthritis (OA) are the most common chronic inflammatory joint diseases, but despite decades of extended research, there are still unmet needs in the treatment of these arthritic disorders, and notably, there are no disease-modifying OA drugs available at present [1]. However, recent evidence indicates that synovial inflammation is also implicated in many of the signs and symptoms of OA, including pain, joint swelling, and effusion. Synovial inflammation in OA resembles that in RA, but the intensity and nature of inflammation may differ between OA and RA [2]. Synovial inflammation is, therefore, a potential target for therapeutic intervention to control joint symptoms, not only in RA but also in OA [2–8].

Methotrexate (MTX) is one of the most frequently used disease-modifying anti-rheumatic drugs, and is considered to act through a mechanism of anti-inflammation [9–12]. Because recent clinical studies have shown that MTX also reduces pain and inflammation in knee OA, MTX has been suggested as a new therapeutic option for OA treatment [13, 14]. However, although MTX is widely used as an effective therapeutic agent in RA, MTX is frequently associated with adverse events such as pneumonitis, liver fibrosis, and myelosuppression, and generally, the main reason for discontinuing MTX is not a lack of efficacy, but its side effects [15, 16]. Therefore, even if MTX were available to treat a chronic disease like OA, there would be serious concerns about its safety aspects. Moreover, an oral treatment such as MTX is not appropriate to treat OA, which is usually regarded as a localized disease, for which the optimal treatment is a therapy applied directly to the affected joint [17, 18].

Intra-articular drug delivery can be useful for treating inflamed joints when a small number of joints are affected, or for joints that do not respond to systemic medications [19, 20]. It has the advantage of targeting drugs to the site of action, thus avoiding systemic side effects. However, the outcomes have not been satisfactory, because drugs administered as an intra-articular formulation rapidly disappear from the joint cavity [19]. To date, various intra-articular drug delivery systems for MTX have been reported [20], including liposomes [21], microspheres [22, 23], and hydrogels [24]. Although there have been some promising preclinical results, none of them have so far been applied in the treatment of arthritic patients. This may be due to the problems common to intra-articular drug delivery systems, such as biocompatibility, tolerability, safety, efficacy, and manufacturing technology [19, 25, 26].

Hyaluronic acid (HA), which is a high molecular weight (MW) polysaccharide composed of repeating subunits of N-acetyl glucosamine and glucuronic acid, is responsible for the viscoelastic properties of synovial fluid, but in OA patients the MW and concentration of HA is decreased [27–30]. In current treatment, intra-articular HA is widely used as viscosupplementation or a symptom-modifying treatment for OA of the knee, because it is generally thought that HA preparations reverse the impaired viscosity and elasticity of synovial fluid that have been caused by the pathologic condition of affected joints and thereby eliminate pain [28–32]. In addition, HA properties — biodegradability, biocompatibility, ease of chemical modification, sufficient safety for long-term clinical use in humans, and an established manufacturing technique — are uniquely suited to address the problems with intra-articular drug delivery mentioned above [33–36].

**Fig. 1:**
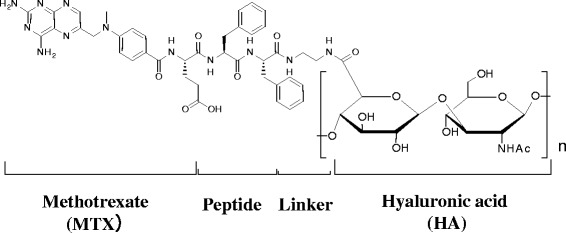
Chemical structure of DK226 [37]. DK226 consists of four parts: MTX, peptide, linker, and HA. MTX binds through its α-carboxylic acid. Peptide and linker are α–Phe–Phe and –NH–(CH_2_)_2_–NH–, respectively

We have therefore been investigating the use of HA as a carrier that will increase the residence time of MTX in affected joints. We believe that HA–MTX conjugates would be a rational combination of these two clinically validated agents, since in such conjugates they each retain their respective biological activity. We reported previously that once-daily intra-articular injections of prototype HA–MTX conjugates showed anti-inflammatory effects in an antigen-induced arthritis model (AIA) [37]. We then optimized the peptide, the linker, the MW, and the binding ratio of MTX to obtain a clinical candidate, DK226 [38] (Fig. 1).

In the present study, we further evaluated DK226 for its mechanisms of action, pharmacokinetics (PK) properties, and preliminary safety and efficacy in two different rat models of arthritis, AIA and collagen-induced arthritis (CIA). Since there are no animal models with a proven track record of modeling the OA synovitis seen in human disease, we used these two inflammatory rat arthritis models to evaluate the potential use of DK226 in the treatment of inflammatory OA. Results obtained in this study suggest the possible use of DK226 as a safe and efficacious agent in arthritic diseases.

## Methods

### Animals

Ten-week-old female DA/Slc rats were obtained from Japan SLC (Shizuoka, Japan). Eleven-week-old male Kbl:JW rabbits were purchased from Oriental Yeast Co., Ltd. (Tokyo, Japan). Five-week-old male LEW/Crj rats were obtained from Charles River Laboratories Japan (Yokohama, Japan). All animal experiments were ethically performed in accordance with the Guidelines for the Care and Use of Laboratory Animals at Chugai Pharmaceutical Co. Ltd, which is accredited by the Association for Assessment and Accreditation of Laboratory Animal Care (AAALAC).

### Preparation of DK226 and MTX-α peptides

DK226 (Fig. 1), MTX-α-phenylalanine (MTX-α-Phe) and MTX-α-phenylalanine-phenylalanine (MTX-α-Phe-Phe) were prepared as described previously [38]. Preparations of DK226 with MW of >1900 kDa and with MTX binding ratio (the percentage of the HA carboxylic acid groups that are replaced by MTX) ranging from 1.9 to 3.8 % were used for the following experiments.

### Cell proliferation assay

Human fibroblast-like synoviocytes (HFLS) (Cell Applicants, Inc., San Diego, CA, USA) or human synovial sarcoma cell line SW982 (ATCC, Manassas, VA, USA) were seeded at 5000 cells/well and cultured in Iscove’s modified Dulbecco’s medium containing 5 % fetal bovine serum and 1× Antibiotic-Antimycotic (Gibco, Life Technologies, Tokyo, Japan) to allow the cells to adhere to the plates. HFLS were stimulated by 10 ng/mL of recombinant human tumor necrosis factor alpha (TNF-α) (R&D Systems, Minneapolis, MN, USA ) and incubated with HA (Suvenyl®, approximately 1900 kDa, Chugai Pharmaceutical Co., Ltd., Tokyo, Japan) or DK226 at 37 °C. SW982 cells were incubated with DK226 or MTX in the presence or absence of 1 μg/mL of anti-CD44 antibody (BU75) (Ancell Corporation, Stillwater, MN, USA) or control IgG2a (RPC 5; Ancell) at 37 °C. After 72-h incubation, ^3^H-deoxyuridine (Moravek Biochemicals, Inc., Brea, CA, USA) was added into the media, and the mixture was cultured for another 48 h (for HFLS) or 4 h (for SW982), followed by radioassay. Radioactivity was calculated as a relative value (% of control), using, as the control, radioactivity in the group of cells cultured without any added test substance.

### MTX release after incubation with rabbit synovial tissue homogenates or synovial fluid

Twelve-week-old male rabbits were subjected to a partial meniscectomy, as described previously [39]. Synovial fluids were collected from the knee joints of the rabbits at 8, 11, 15, 18, 25, and 29 days after the operation and then combined and stored at −80 °C until use. Twenty-nine days after the operation, the animals were euthanized by exsanguination under ether anesthesia, and the synovial tissues were dissected, weighed, and homogenized in a 4-fold volume of 0.05 mol/L Tris (hydroxymethyl) aminomethane–hydrochloride (Tris–HCl) buffer (pH 8.0) containing 0.138 mol/L sodium chloride (NaCl) and 0.0027 mol/L potassium chloride (KCl). The homogenates were combined and stored at −80 °C until use. The protein contents of the combined synovial tissue homogenates and the combined synovial fluids were determined by Bradford protein assay. Next, 40 μg/mL of DK226 or 10 μg/mL of MTX-α-Phe (reference compound to measure metabolic activity) was incubated with or without synovial tissue homogenate or synovial fluids (500 μg protein/mL) in 0.1 mol/L acetate buffer (pH 4.0) or 0.1 mol/L phosphate buffer (pH 7.4) at 37 °C for 3, 8, and 24 h. At the end of each incubation period, 100 μL of the reaction mixture was collected and mixed with 1 mL of ethanol. After evaporation of the solvent under nitrogen, the residues were dissolved in 100 μL of 10 mmol/L ammonium acetate in 10 % acetonitrile. Concentrations of MTX, MTX-α-Phe, and MTX-α-Phe-Phe were measured by liquid chromatography/quadrupole time-of-flight mass spectrometry (Q-TOF Ultima API; Waters, Milford, MA, USA). To assess the metabolic stability of MTX-α-Phe, the percent peak area of intact MTX-α-Phe and MTX released from MTX-α-Phe were monitored by liquid chromatography with UV detection (LC-UV) at 215 nm (Magic 2002; AMR Inc., Tokyo, Japan).

### Induction and treatment of AIA

Six-week-old male LEW/Crj rats were immunized as previously described [38]. On the day of arthritis induction (day 0), rats were treated with a single intra-articular injection of 50 μL of modified bovine serum albumin (mBSA) (Merck, Darmstadt, Germany) aqueous solution (2 mg/mL) into the right knee joint. The left knee joint was untreated and served as the control. MTX, at a dose of 1.25 mg/kg was administered orally five times a week from 7 days before inducing arthritis (day −7). DK226 or vehicle (phosphate-buffered saline, PBS) was intra-articularly administered into the right knee joint in an amount of 50 μL (0.5 mg HA-equiv, 30 nmol MTX-equiv) on day −7, day −1, day 7, and day 14. In a separate experiment, HA (0.5 mg), free MTX (25 nmol), a mixture of HA (0.5 mg) and MTX (25 nmol), or DK226 (0.5 mg HA-equiv, 25 nmol MTX-equiv) was intra-articularly administered into the right knee joint on day–7, day −1, and day 7. Knee joint swelling was assessed as previously described [38].

### Assessment of hematological toxicity in AIA

Blood samples were collected from the tarsal veins of AIA rats at 24 h after treatment with oral MTX or DK226 on day14, and were assayed for hematological analysis. White blood cells, red blood cells, hemoglobin, hematocrit, and platelet values were measured with an automatic cell counter (F-820) (Sysmex, Kobe, Japan).

### Measurements of plasma MTX in AIA

Serial blood samples were collected from AIA rats on day 14 at 0 h (immediately before treatment) and at 0.25, 0.5, 1, 2, 4, 8, and 24 h after treatment with oral MTX, and at 4, 8, 24, 48, and 72 h after treatment with DK226. Plasma MTX, MTX-α-Phe, and MTX-α-Phe-Phe levels were measured by liquid chromatography-tandem mass spectrometry (LC-MS/MS). Briefly, 20 μL of 12.5 ng/mL of internal standard and 1 mL of 1 % (v/v) formic acid in acetonitrile was added to 100 μL of rat plasma and stirred for 2 min. After centrifugation (1710 × g, 5 min, 4 °C), the supernatant was collected and dried under nitrogen. The residues were dissolved in 60 μL of 4 % (w/v) trichloroacetic acid, and the supernatant was analyzed by LC-MS/MS (API 4000, AB SCIEX, Framingham, MA, USA). Lower limits of quantification (LLOQ) were 50 pg/mL for MTX and 100 pg/mL for MTX-α-Phe and MTX-α-Phe-Phe. PK parameters [time to reach maximum plasma concentration (T_max_)_,_ maximum plasma concentration (C_max_), area under the plasma concentration-time curve calculated from time zero to infinity (AUC_inf_)_,_ and terminal phase elimination half-life (t_1/2_)] were calculated by a non-compartmental model.

### Induction and treatment of CIA

Eleven-week-old female DA/Slc rats were immunized intradermally at four sites on the back with 0.3 mg of bovine type II collagen (Collagen Research Center, Tokyo, Japan) emulsified with an equal volume of incomplete Freund’s adjuvant (Difco, Detroit, MI, USA) on day 0. Vehicle (saline), HA (0.5 mg), or DK226 (0.5 mg HA-equiv, 26 nmol MTX-equiv) was administered into the right knee joint in an amount of 50 μL at 5-day intervals from day 0 to day 20. The left knee joint was untreated. Three animals were not immunized and served as the control (Normal). Knee joint swelling was assessed by measuring the width of each knee joint with calipers on days 0, 5, 10, 13, 15, 17, 20, and 23. On day 23, animals were euthanized by exsanguination under anesthesia, and the right knee joints were harvested for histological evaluation.

### Histological evaluation

The right knee joints of the CIA rats were fixed overnight in 20 % neutral-buffered formalin at room temperature. The samples were dehydrated and embedded in paraffin, and then cut on the midsagittal plane to examine the synovium and cut on the longitudinal plane to examine the lateral femoral condyle. Sections (3 μm thick) were stained with hematoxylin and eosin or safranin O/fast green and evaluated according to the following semiquantitative grading scale: 0 = no pathological changes; 1 = minimal (minimal changes, or lesions involving <25 % of the whole section); 2 = slight (obvious changes, or lesions affecting 25–50 % of the whole section); 3 = moderate (relatively severe changes, or lesions involving 50–75 % of the whole section); 4 = severe (very severe changes, or lesions affecting >75 % of the whole section). Synovium was examined for exudation in the joint cavity, fibrosis, granulation tissue formation, proliferation of synovial cells, inflammatory cell infiltration, and edema. The lateral femoral condyle was assessed for decrease in safranin O staining, osteophyte formation, destruction of cartilage, destruction of cortical bone, and pannus formation.

### Statistical analysis

Statistical significances were estimated by unpaired Student’s *t* test and Wilcoxon rank sum test using a statistical software package (SAS) version 6.12; SAS Institute Japan, Tokyo, Japan) with the significance level set to 5 %.

## Results

### Anti-proliferative effects on synovial cells

**Fig. 2:**
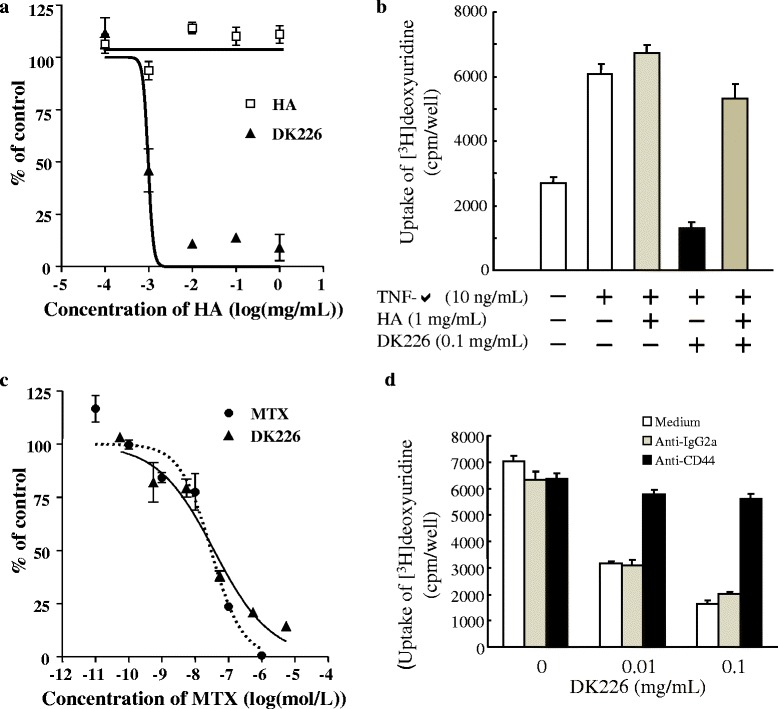
Effect of DK226 on proliferation of human synovial fibroblast-like cells (HFLS) and of human synovial sarcoma cell line SW982. **a** Inhibition of tumor necrosis alpha (TNF-α)-induced proliferation of HFLS by hyaluronic acid (HA) or DK226 at increasing, equivalent HA concentrations. **b** Effect of exogenously added HA on the anti-proliferative effect of DK226 in HFLS. **c** Inhibition of proliferation of SW982 by methotrexate (MTX) or DK226 at increasing, equivalent MTX concentrations. **d** Effect of exogenously added anti-CD44 antibody (BU75) and anti-IgG2a (control antibody) on the anti-proliferative effect of DK226 in SW982. Values are means and standard error of the mean (SEM) (n = 4)

To verify the biological activity of DK226 and to confirm whether the conjugate binds to HA receptors, we examined the effects of DK226 on proliferation of synovial cells with or without exogenously added HA or anti-CD44 antibody. TNF-α-induced proliferation of HFLS cells was inhibited by DK226, but not by HA (up to 1 mg/mL), in a dose-dependent manner (Fig. 2a). However, the inhibition by DK226 (0.1 mg/mL) was reversed by adding exogenous HA (1 mg/mL) (Fig. 2b). This implies the involvement of a HA receptor, such as CD44, in the anti-proliferative effects induced by DK226. In preliminary experiments, the inhibitory effect of DK226 was rescued by adding anti-CD44 antibody, but anti-CD44 antibody itself stimulated TNF-α-induced proliferation of HFLS cells. To address this problem, we used a synovial cell line, SW982, in which anti-CD44 antibody had no effect on the proliferation of the cells. Cell proliferation of SW982 was also inhibited by DK226 in a dose-dependent manner; the potency of DK226 was comparable to that of free MTX (Fig. 2c). The inhibitory effect of DK226 (0.1 and 0.01 mg/mL) in SW982 was reversed by adding anti-CD44 antibody, but not by adding control IgG2a antibody (Fig. 2d).

### Release of MTX from DK226

In DK226, MTX binds to the HA backbone via a peptide (α-Phe-Phe) and an alkyl linker (Fig. 1). We previously demonstrated that MTX-α peptides were susceptible to cleavage by cathepsins to release MTX at acidic pH [38]. Since cathepsins are abundantly expressed in synovial tissue and synovial fluid [40], we expected that MTX would be released from DK226. To examine this possibility, DK226 was incubated with synovial tissue homogenate or synovial fluid from the knee of a rabbit OA model [39]. As shown in Table 1, MTX was released from DK226 in the presence of synovial tissue or synovial fluid at pH 4.0, but not at pH 7.4. MTX-α peptides were detected in the culture media when incubated with synovial fluid at pH 4.0, but neither MTX nor MTX-α peptides were found when incubated in buffer alone (pH 4.0 or pH 7.4) (data not shown). The percentage of reference compound (MTX-α-Phe) after 24-h incubation with synovial tissue homogenate and with synovial fluid was 100 % for both at pH 7.4 (after 3-h incubation), or 0 % and 80.1 % respectively at pH 4.0 (Table 1), whereas the percentage in buffer alone was 100 % at both pH 4.0 and pH 7.4 (data not shown).

**Table 1:** Release of MTX and MTX-α peptides from DK226

Treatment	pH	Released concentration (ng/mL) MTX MTX-α-Phe MTX-α-Phe-Phe			% of released MTX	% peak area of MTX-α-Phe after 24-h incubation
Synovial tissue homogenate	4.0	8.82	BLQ^b^	BLQ^c^	1.2	0^e^
	7.4	BLQ^a^	BLQ^b^	BLQ^c^	0	100
Synovial fluid	4.0	1.14	2.02 (1.53)^d^	1.29 (0.783)^d^	0.5	80.1
	7.4	BLQ^a^	BLQ^a^	BLQ^a^	0	100

DK226 was incubated in rabbit synovial tissue homogenate or synovial fluid at pH 4.0 or pH 7.4 for 24 h at 37 °C. Release of MTX and MTX-α peptides was measured by LC/Q-TOF-MS. MTX-α-Phe was used as a reference compound to assess the metabolic stability, and the percent peak area of intact MTX-α-Phe and MTX released from MTX-α-Phe were monitored by LC-UV

*MTX* methotrexate, *Phe* phenylalanine, *BLQ* below lower limit of quantification

^a^BLQ (below LLOQ); <1 ng/mL

^b^BLQ; <5 ng/mL

^c^BLQ; <10 ng/mL

^d^MTX-equivalent concentration

^e^Percent peak area of MTX-α-Phe after 3-h incubation

### Efficacy and safety of DK226 in AIA

**Fig. 3:**
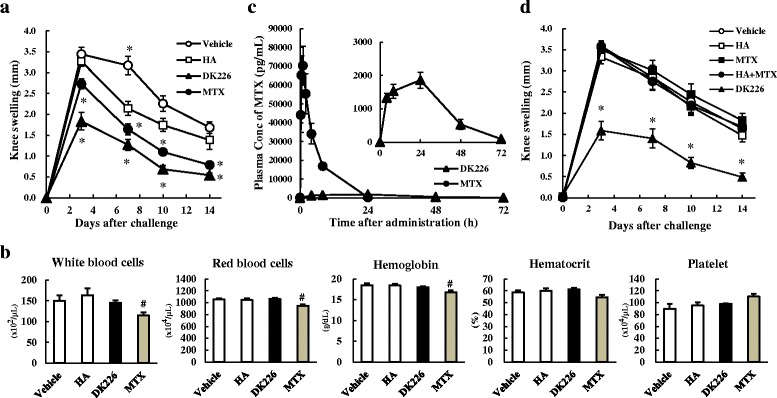
Effect of DK226 on antigen-induced arthritis (AIA) in rat knee joints. **a** Effects of intra-articular hyaluronic acid (HA) (0.5 mg), DK226 (0.5 mg HA-equiv, 30 nmol MTX-equiv), or oral methotrexate (MTX) (1.25/mg/kg) on knee swelling. **b** Hematological parameters of AIA treated with intra-articular DK226 or oral MTX. Blood samples were collected at 24 h after treatment on day 14. **c** MTX plasma profiles following intra-articular DK226 or oral MTX on day 14. Inserted figure is a magnified view of the MTX plasma concentration after intra-articular DK226. Plasma concentrations of MTX were measured with liquid chromatography-tandem mass spectrometry (LC-MS/MS). **d** Effects on knee swelling of intra-articular injections of HA (0.5 mg), MTX (25 nmol), mixture of HA (0.5 mg) and MTX (25 nmol), and DK226 (0.5 mg HA-equiv, 25 nmol MTX-equiv). Values are means and standard error of the mean (SEM) (n = 7 in **a** and **b**; n = 4 in **c**; n = 6–8 in **d**). *P < 0.01: significantly different from HA group (Student’s *t* test). ^#^ *P* < 0.05: significantly different from Vehicle group (Student’s *t* test)

A significant reduction in knee swelling was observed with once-weekly intra-articular DK226, but HA had only a marginal effect on day 7 (Fig. 3a). Efficacy of DK226 was comparable to that of oral MTX. To compare the safety of DK226 and oral MTX, we examined the effects of these two treatments on hematological parameters in AIA rats at 24 h after treatment with oral MTX or DK226 on day 14. Although oral MTX reduced white blood cells (*P* < 0.05), red blood cells (*P* < 0.05), hemoglobin (*P* < 0.05), and hematocrit values, and increased platelets, DK226 did not show any hematological abnormalities (Fig. 3b). The MTX plasma profiles after DK226 or oral MTX are shown in Fig. 3c, and their PK parameters are summarized in Table 2.

**Table 2:** Pharmacokinetics parameters for MTX plasma profiles of antigen-induced arthritis after treatment with oral MTX or DK226

Compound	Dose	T_max_ (h)	C_max_ (pg/mL)	AUC_inf_ (ng · h/mL)	t_1/2_ (h)
MTX	1.25 mg/kg, oral^a^	0.9 ± .0.3	72,600 ± 10,000	365 ± 37	2.78 ± 0.18
DK226	50 μL/body, intra-articular^b^	20.0 ± 8.0	1900 ± 180	68.4 ± 6.1	11.2 ± 0.4

AIA rats were dosed with oral MTX (five times a week, day −7 to day 14) or intra-articular DK226 (weekly, day −7 to day 14) to produce a comparable suppression of knee joint swelling. Serial blood samples were collected from AIA rats on day 14 at 0 h (immediately before treatment) and at 0.25, 0.5, 1, 2, 4, 8, and 24 h after treatment with oral MTX, and at 4, 8, 24, 48, and 72 h after treatment with DK226. Plasma concentrations of MTX were then determined by LC-MS/MS. PK parameters were calculated by a non-compartmental model

*MTX* methotrexate, *T*_*max*_ time to reach maximum plasma concentration, *C*_*max*_ maximum plasma concentration, *AUC*_*inf*_ area under the plasma concentration-time curve calculated from time zero to infinity, *t*_*1/2*_ terminal phase elimination half-life

^a^The dose of MTX administered; 375 μg

^b^The dose of MTX injected; 13.6 μg

The plasma MTX concentration peaked at 72,600 pg/mL at 0.9 h after oral administration of MTX, and returned to the control level by 24 h (AUC_inf_: 365 ng∙h/mL, t_1/2_: 2.78 h). For animals treated with DK226, the plasma MTX rose gradually to a level of 1900 pg/mL at 20–24 h after articular injection, and a low plasma concentration was maintained up to 72 h (AUC_inf_: 68.4 ng∙h/mL, t_1/2_: 11.2 h). MTX-α-Phe and MTX-α-Phe-Phe concentrations were below the LLOQ (<100 pg/mL).

To demonstrate the importance of the conjugation between HA and MTX in DK226, the effects of weekly intra-articular injections of DK226, free MTX, free HA, and a mixture of HA and MTX on AIA were compared. As shown in Fig. 3d, AIA rats treated with DK226 showed a significant reduction in knee swelling. In contrast, free MTX, free HA, or a mixture of HA and MTX had no or little effect on the joint swelling.

### Inhibitory efficacy in CIA

**Fig. 4:**
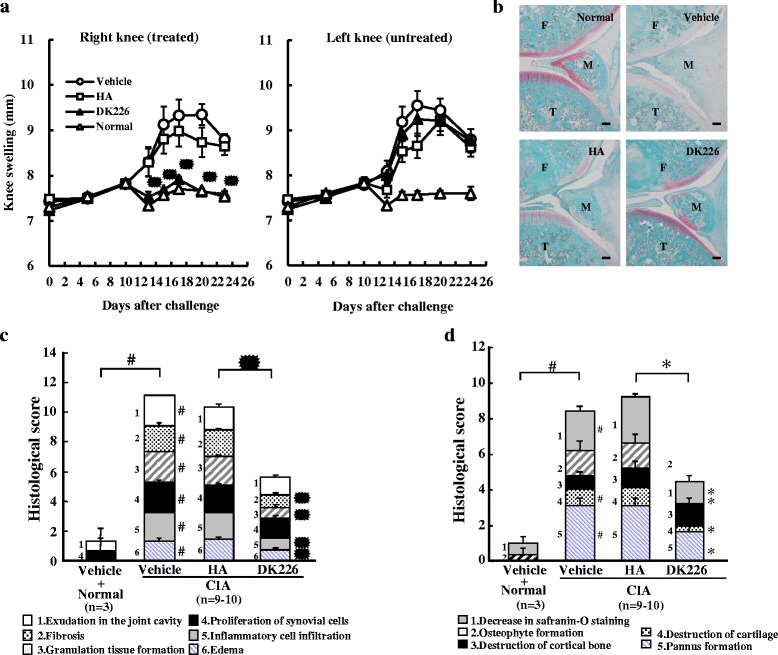
Effect of DK226 on collagen-induced arthritis (CIA) in rat knee joints. **a** Effect of intra-articular hyaluronic acid (HA) (0.5 mg) or DK226 (0.5 mg HA-equiv, 26 nmol MTX-equiv) on knee swelling of the right knee (treated) shown on the *left panel*, and the effect in the left knee (untreated) shown on the *right panel*. **b** Histopathology of the right knee joints after treatment with intra-articular HA or DK226. Sections were stained with safranin O/fast green. *T* tibia, *M* meniscus, *F* femur. Scale bars indicate 100 μm. **c** and **d** Histologic analysis of synovial tissue (**c**) and lateral condyle of the femur (**d**). Values are means and standard error of the mean (SEM) (n = 3–10 in **a**, **c** and **d**: n = 3, Normal + vehicle; n = 9–10, CIA + vehicle, HA or DKA226). ^#^ *P* < 0.05: significantly different from Normal + vehicle group (Wilcoxon rank sum test). *P < 0.05: significantly different from HA group (Wilcoxon rank sum test)

To investigate the effect of DK226 in a different arthritic model, we examined the efficacy of the conjugate in CIA, in which significant swellings have been induced in the right and left knee joints. From day 0 to day 23, rats injected in the right knee joint with DK226 showed a significant reduction in swelling in the right knee joint, but not in the uninjected contralateral left knee joint. Rats injected with HA did not show any difference in the right and left knee swellings (Fig. 4a). In a separate experiment, knee swellings were also suppressed in CIA on day 15 after DK226 had been administered on day 3 and day 10 (data not shown). Histopathological examination of the right knee joints showed that edema, inflammatory cell infiltration, proliferation of synovial cells, articular cartilage erosion, and a decrease in safranin O staining were prevented or ameliorated by DK226 (Fig. 4b). In contrast, vehicle or HA had little or no effect on these arthritic changes (Fig. 4b). Fibrosis, granulation tissue formation, inflammatory cell infiltration, and edema in synovium (Fig. 4c), and decrease in safranin O staining, osteophyte formation, destruction of cortical bone, and pannus formation in the lateral condyle of the femur (Fig. 4d) were significantly reduced in the DK226-treated group as compared to the HA-treated group.

## Discussion

The present study clearly demonstrated that intra-articular DK226 showed efficacy comparable to that of oral MTX while avoiding the systemic risks of MTX. Rats receiving oral MTX showed hematological toxicities, but none of the animals treated with DK226 exhibited any adverse signs (Fig. 3b). In a preliminary toxicity study in normal rats, once-a-week injections of DK226 to the right and left knee joints for a month resulted in no histopathological abnormalities in the injected joints, and displayed no systemic toxicological abnormalities in hematological, serum biochemical, and histopathological examinations (data not shown). Our study also demonstrates that the total amount of MTX administered can be dramatically reduced by using DK226 rather than oral MTX. Although 1.25 mg/kg oral MTX given five times a week (1875 μg/week) was required to exert sufficient efficacy in AIA, a once-weekly intra-articular injection of DK226 (corresponding to around 13.6 μg/week) provided efficacy equivalent to that of oral MTX in the same model. This difference appears to be attributable to the difference in the PK properties of the two agents. PK analysis demonstrated that the C_max_ and the AUC_inf_ following oral administration of MTX were approximately 40 times and 5 times those of DK226 (Table 2). We then examined the effects of intra-articular injections of DK226, HA, free MTX, and a mixture of HA and free MTX on AIA. Interestingly, intra-articular injection of DK226 (0.5 mg HA-equiv, 25 nmol MTX-equiv) produced a significant reduction in knee swelling, whereas the same amount of HA (0.5 mg), free MTX (25 nmol), or a mixture of HA (0.5 mg) and MTX (25 nmol) showed no effect on the model (Fig. 3d). It has been reported that intra-articular injection of MTX disappears very rapidly from the knee joints and produces a rapid increase in serum MTX (18–20). Radiolabeled HAs injected into the knee joints of normal rabbits declined far more slowly, with average half-lives of less than 24 h [41], and measurable radioactivity within the synovial layer and the articular cartilage was detected up to at least 48 h after injection [41, 42]. These results, combined with the PK parameters described before (Table 2), indicate that intra-articular injection of DK226 is cleared from the joint cavity much more slowly than that of free MTX. In other words, covalent conjugation with HA, but not a simple HA and MTX mixture, can prolong the residence time of MTX in the joint cavity.

In DK226, MTX and HA are linked with a peptide linker (Fig. 1), which is susceptible to cleavage by lysosomal enzymes including cathepsins [38]. DK226 showed anti-proliferative effects on HFLS and SW982 cells, and these effects were reversed by adding exogenous HA or anti-CD44 antibody (Fig. 2a–d). We previously demonstrated that prototype HA–MTX conjugates lacking peptide chains failed to show an inhibitory effect on HFLS proliferation [37]. The receptors recognizing HA, such as CD44 and receptor for hyaluronan-mediated motility, are expressed on the surface layer of a wide range of cells, including inflammatory cells, synovial cells, and chondrocytes [43–46]. A number of studies have shown that HA receptors, especially CD44, are responsible for the receptor-mediated cellular uptake and degradation of HA [47, 48]. As shown in Table 1, when DK226 is incubated with rabbit synovial tissue homogenate or synovial fluid, release of MTX from DK226 occurs at pH 4.0 but not at pH 7.4. Although the pH of the synovial fluid from knee joints of AIA and CIA rat models could not be determined due to their small volume, the pH of synovial fluid in arthritis is reported to be neutral in RA and OA patients [49] or slightly acidic in a rabbit OA model (normal: pH 7.32, OA: pH 6.97) [50]. We therefore speculate that DK226 is distributed in the synovium and can retain a sufficient amount of MTX until it is internalized and cleaved by lysosomal enzymes to release the pharmacologically active form of MTX. However, we cannot exclude the possibility that, in vivo, the conjugate is degraded in tissues other than the synovium and degraded by other enzymes in the affected joints. Taken together, our results suggest that DK226 can reduce the side effects caused by current MTX therapy by lowering systemic exposure, by prolonging retention time in the joint, and possibly by HA receptor–mediated internalization and degradation.

Another unique feature of DK226 is that the HA in DK226 has a high MW (>1900 kDa) comparable to native HA in the joint. Thus, the conjugate is expected to act not only as a carrier of MTX, but also to behave like native HA. Although the mode of action of HA has not been fully elucidated, intra-articular injection of HA is used worldwide as a symptom-modifying treatment for OA of the knee [28–32], and some HA products are used in Japan for treating RA of the knee [51]. Reports have suggested that the beneficial effects of HA in OA of the knee may depend on its rheological properties and MW, and that high MW HA tends to remain in the joint cavities longer than low MW HA [29–32]. Low MW HA has different and more diverse biological activities than high MW HA [29, 30]. For these reasons, control of MW is a prerequisite for the design and synthesis of DK226 [38]. To the best of our knowledge, DK226 is the first example of a HA–drug conjugate with a MW comparable to that of native HA.

OA has long been considered a degenerative disease, but a number of studies have suggested that pain in the disease is also caused by synovial inflammation [2–6]. The differences in synovial inflammation in OA and RA were largely thought to be quantitative rather than qualitative; angiogenesis [52], T cell accumulation [53] and synovial cell proliferation and interleukin (IL)-1β expression [54] were higher in RA than in OA. But, qualitative differences have also been observed in immune cell filtration and cytokine production between synovial inflammation in OA and RA. It is also reported that synovial inflammation in OA is located close to a cartilage lesion, in contrast to RA, which shows diffuse distribution [55]. A recent randomized, placebo-controlled study of MTX demonstrated that MTX reduced pain and improved synovitis in patients with symptomatic knee OA [13]. An open-label study of MTX also demonstrated its analgesic efficacy in OA of the knee [14]. These findings may indicate that there is a common underlying mechanism contributing to synovial inflammation in RA and OA, and that MTX might be a therapeutic option in the treatment of certain types of OA [3, 13, 14]. However, since OA is not a life-threatening disease, treatments are required to have a lower level of risk than in RA. Moreover, OA, which limits disability to weight-bearing joints, is not a systemic disease; therefore, safe and effective means for reducing the systemic toxicity of MTX are more appropriate for the treatment of OA. In addressing the particular needs of OA treatment, the safer toxicity profiles and local administration route of DK226 provide a new option. In CIA, an injection of DK226 in the right knee produced a significant reduction in joint swelling in the DK226-treated knee, but not in the contralateral untreated knee. The fact that the conjugate has no influence on uninjected joints may also indicate that DK226 has a favorable profile as a local therapy for treating inflamed joints resistant to systemic medications or persistent inflammatory monoarthritis [19].

HA products currently available to treat knee OA generally reduce pain but do not fully control inflammation. In the present study, we demonstrated the anti-arthritic efficacy of DK226 in rat AIA and CIA at a dose that is equivalent to the clinical dose of conventional HA products used to treat knee OA. Although HA alone had little or no effect on synovial inflammation, DK226 reduced inflammation significantly in both models. Because the mechanisms of action of HA and MTX are distinct, additive or synergistic effects of the two agents can be expected with DK226 in clinical use. In fact, the mechanism of action of HA itself is far from clear. Recent reports show that, although HA disappears from synovial fluid within several days, the effect of intra-articular HA products lasts for over 4 weeks [31], and the possibility that intra-articularly injected HA eliminates pain by a mechanism other than by simply improving the viscoelasticity of synovial fluid (as described above) has been suggested [30–32, 56]. Further examinations are needed to show whether a reduced dose of DK226 or a less frequent treatment regimen is also effective.

Even though MTX is widely used for the treatment of RA, little is known about its direct effect on cartilage. Studies into the effects of MTX on chondrocyte function have failed to show any significant effect on cartilage metabolism in vitro or on cartilage degeneration in normal animals or in rabbit OA models in vivo [57–59]. Although MTX may prevent cartilage breakdown to some extent in animal models or in humans, this is probably due to a reduction in synovial inflammation rather than to its direct effects on cartilage, since synovial inflammation in OA is considered to be secondary to mechanical damage of cartilage [60]. In preliminary experiments, we sought to examine whether synovial inflammation could be assessed in a rat model with OA surgically induced by medial meniscectomy in the knee. While cartilage degeneration was observed, the degree of synovial inflammation induced in the model was too mild to measure reliably (data not shown). A low degree of synovial inflammation was also reported in a surgical OA model in mouse [61], and a collagenase-induced OA model in rabbit [39]. We therefore examined the effects of DK226 in AIA and CIA to demonstrate that the anti-inflammatory effect of MTX was preserved after conjugation with HA, and consequently fulfilled the primary aim of this study, which was to suggest the potential of DK226 as an anti-arthritic drug. However, AIA and CIA, which share some clinical and pathological features with RA in humans [62, 63], are widely used to explore disease mechanisms of RA and to predict the clinical efficacy of a candidate RA drug, rather than for OA. To better evaluate the potential of DK226 as an anti-OA drug, the efficacy of DK226 should be tested in animal models of OA that involve synovial inflammation resembling that in human OA. However, to date there are no animal models that have the same characteristics as inflammatory OA in humans, and further studies are warranted to establish new animal models of OA that are valid not only for DK226 but for other new drugs for inflammatory OA.

## Conclusions

DK226 is a new type of high molecular weight HA product with anti-inflammatory efficacy. DK226 can exert the effect of the two conjugated agents, MTX and HA, both of which have long been used to treat arthritis. This could be a novel approach for the treatment of patients with arthritic disorders, including inflammatory OA.

## Abbreviations

AIA: antigen-induced arthritis
AUC_inf_: area under the plasma concentration-time curve calculated from time zero to infinity
BSA: bovine serum albumin
CIA: collagen-induced arthritis
C_max_: maximum plasma concentration
HA: hyaluronic acid
HCl: hydrochloride
HFLS: human fibroblast-like synoviocytes
IL: interleukin
KCl: potassium chloride
LC: liquid chromatography
LCMS/MS: liquid chromatography-tandem mass spectrometry
LLOQ: lower limit of quantification
MTX: methotrexate
MW: molecular weight
NaCl: sodium chloride
OA: osteoarthritis
PBS: phosphate-buffered saline
Phe: phenylalanine
PK: pharmacokinetics
Q-TOF: quadrupole time-of-flight
RA: rheumatoid arthritis
t_1/2_: terminal phase elimination half-life
SEM: standard error of the mean
T_max_: time to reach maximum plasma concentration
TNF-α: tumor necrosis factor alpha
Tris: Tris (hydroxymethyl) aminomethane
