# Erratum to: Novel hyaluronic acid–methotrexate conjugate suppresses joint inflammation in the rat knee: efficacy and safety evaluation in two rat arthritis models

**DOI:** 10.1186/s13075-016-1029-7

**Published:** 2016-05-31

**Authors:** Tatsuya Tamura, Yoshinobu Higuchi, Hidetomo Kitamura, Naoaki Murao, Ryoichi Saitoh, Tadashi Morikawa, Haruhiko Sato

**Affiliations:** Research Division, Chugai Pharmaceutical Co., Ltd., 1-135 Komakado, Gotemba, Shizuoka 412-8513 Japan; New Business Planning Department, Denka Co., Ltd., 2-1-1 Nihonbashi-Muromachi, Chuo-ku, Tokyo, 103-8338 Japan

After publication of this article [1] it was noticed that Figs. 2 and 4 (seen below) contained incorrect characters. The corrected figures can be seen below and the original article has also been updated to reflect this.

**Fig. 2:**
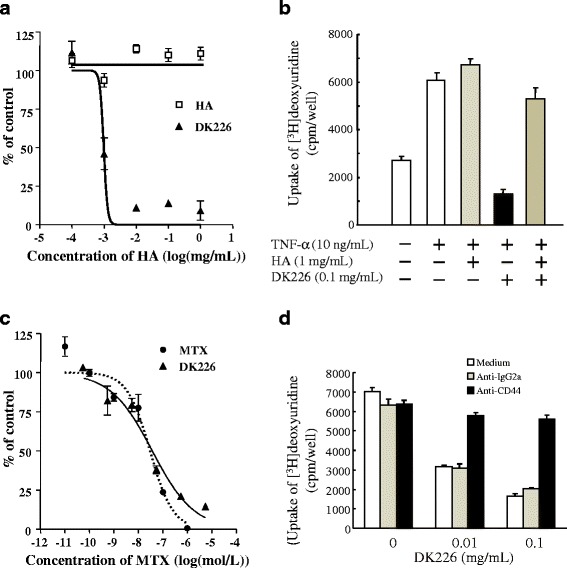
Effect of DK226 on proliferation of human synovial fibroblast-like cells (HFLS) and of human synovial sarcoma cell line SW982. **a** Inhibition of tumor necrosis alpha (TNF-α)-induced proliferation of HFLS by hyaluronic acid (HA) or DK226 at increasing, equivalent HA concentrations. **b** Effect of exogenously added HA on the anti-proliferative effect of DK226 in HFLS. **c** Inhibition of proliferation of SW982 by methotrexate (MTX) or DK226 at increasing, equivalent MTX concentrations. **d** Effect of exogenously added anti-CD44 antibody (BU75) and anti-IgG2a (control antibody) on the anti-proliferative effect of DK226 in SW982. Values are means and standard error of the mean (SEM) (*n* = 4)

**Fig. 4:**
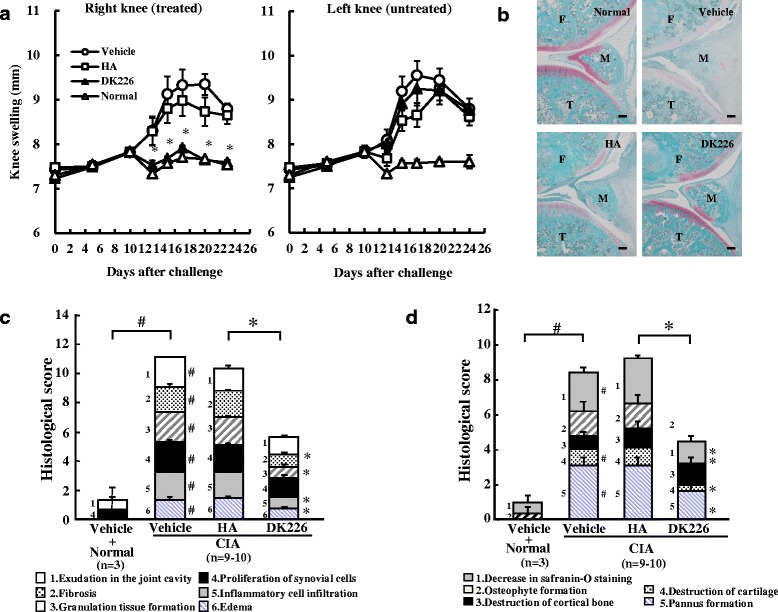
Effect of DK226 on collagen-induced arthritis (CIA) in rat knee joints. **a** Effect of intra-articular hyaluronic acid (HA) (0.5 mg) or DK226 (0.5 mg HA-equiv, 26 nmol MTX-equiv) on knee swelling of the right knee (treated) shown on the *left panel*, and the effect in the left knee (untreated) shown on the *right panel*. **b** Histopathology of the right knee joints after treatment with intra-articular HA or DK226. Sections were stained with safranin O/fast green. *T* tibia, *M*meniscus, *F* femur. Scale bars indicate 100 μm. **c** and **d** Histologic analysis of synovial tissue (**c**) and lateral condyle of the femur (**d**). Values are means and standard error of the mean (SEM) (*n* = 3–10 in **a**, **c** and **d**: *n* = 3, Normal + vehicle; *n* = 9–10, CIA + vehicle, HA or DKA226). ^#^ *P* < 0.05: significantly different from Normal + vehicle group (Wilcoxon rank sum test). *P < 0.05: significantly different from HA group (Wilcoxon rank sum test)
